# The dual role of ferroptosis in anthracycline-based chemotherapy includes reducing resistance and increasing toxicity

**DOI:** 10.1038/s41420-023-01483-1

**Published:** 2023-06-21

**Authors:** Jiazheng Zhao, Ning Zhang, Xiaowei Ma, Ming Li, Helin Feng

**Affiliations:** 1 Department of Orthopedics, The Fourth Hospital of Hebei Medical University, 12 Health Road, Shijiazhuang, Hebei 050011 China; 2 Department of Cardiology, The Fourth Hospital of Hebei Medical University, 12 Health Road, Shijiazhuang, Hebei 050011 China; 3 Departments of Orthopedics, National Cancer Center/National Clinical Research Center for Cancer/Cancer Hospital, Chinese Academy of Medical Sciences and Peking Union Medical College, No. 17 Nanli, Panjiayuan, Chaoyang District, Beijing, 100021 China; 4 Department of Orthopedics, The Second Hospital of Hebei Medical University, 215 Heping Road, Shijia-zhuang, Hebei China

**Keywords:** Cancer, Drug discovery

## Abstract

In conjunction with previous studies, we have noted that ferroptosis, as an emerging mode of regulated cell death (RCD), is intimately related to anthracycline pharmacotherapy. Not only does ferroptosis significantly modulate tumour resistance and drug toxicity, which are core links of the relevant chemotherapeutic process, but it also appears to play a conflicting role that has yet to be appreciated. By targeting the dual role of ferroptosis in anthracycline-based chemotherapy, this review aims to focus on the latest findings at this stage, identify the potential associations and provide novel perspectives for subsequent research directions and therapeutic strategies.

## Facts

1. Ferroptosis is characterized by iron-dependent reactive oxygen species (ROS) deposition and is closely associated with anthracycline-based chemotherapy.
2. Not only does ferroptosis significantly modulate tumour resistance and drug toxicity, which are core links of the relevant chemotherapeutic process, but it also appears to play a conflicting role that has yet to be appreciated.
3. Targeted ferroptosis is a potential effective means to regulate the action of anthracyclines.

## Open Questions

1. What is the specific mechanism by which ferroptosis regulates the action of anthracyclines?
2. What is the dual role played by ferroptosis in anthracycline chemotherapy?
3. How to achieve improved efficacy and reduced side effects during anthracycline chemotherapy by regulating ferroptosis?

## Introduction

Chemotherapy-based treatment is currently the first-line regimen for the vast majority of tumours [1]. Anthracyclines and their related derivatives, including doxorubicin (DOX), are generally prevalent and highly effective anticancer drugs that have been widely applied for the management of various malignancies, including breast cancer, haematologic diseases, and sarcomas, as the cornerstone of multiple chemotherapy regimens [2, 3]. Nevertheless, not all tumours exhibit a high level of sensitivity to the cytotoxic effect of anthracyclines, and tumour cells that are initially chemotherapy-sensitive may become increasingly resistant to the drugs as treatment progresses [4, 5]. Therefore, feasible attenuation of tumour resistance to anthracyclines is essential to enhance patient survival and improve patient prognosis. Meanwhile, the side effects that anthracyclines bring cannot be ignored; the most prominent side effect is anthracycline-induced cardiotoxicity (AIC), which is characteristically cumulative and irreversible [6, 7]. In addition, anthracyclines can also mediate varying degrees of hepatotoxicity and nephrotoxicity [8], and these drug toxicities significantly restrict the dose and scope of anthracycline clinical applications. Since the corresponding mechanisms underlying anthracycline pharmacotherapy are not exactly clarified, how to reduce side effects while diminishing chemotherapy resistance remains an urgent question to be addressed.

Ferroptosis, an emerging type of regulated cell death (RCD) with unique biological features [9, 10], has been identified to play a critical role in the origination, development and treatment of a wide range of diseases including both malignant tumours and the cardiovascular system [11]. At present, as far as anthracyclines are concerned, practically all studies have uniformly indicated that the occurrence of ferroptosis facilitates the attenuation of chemotherapy resistance in tumour cells to promote the efficacy of anthracyclines [12, 13]. Meanwhile, a growing number of studies have revealed that the inhibition of ferroptosis mediates the diminution of anthracycline toxicities, leading to protection of corresponding normal target organs [14]. Therefore, it appears unviable to simultaneously achieve satisfactory outcomes for both tumour tissue and normal tissue by interfering with ferroptosis mechanisms during anthracycline therapy, as ferroptosis seems to play a conflicting role. However, few studies have previously investigated the role of ferroptosis in both chemotherapy resistance and drug toxicity; here, we plan to focus on and further explore the potential conflicting relationships.

Against this background, the present review focuses on the dual identity of ferroptosis in the treatment of anthracyclines and does not elaborate on what has been frequently previously reported and widely known; rather, this review focuses on the latest findings at this stage, identifies potential associations and provides novel perspectives for subsequent research directions and therapeutic strategies.

### The role of ferroptosis in anthracycline activity

**Fig. 1:**
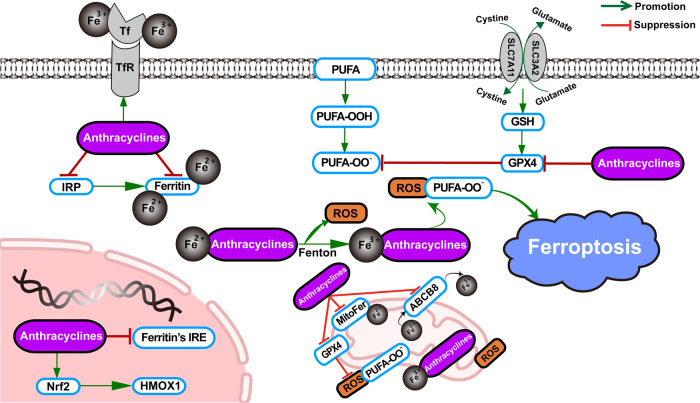
Anthracycline-mediated ferroptosis. Through multiple pathways, anthracyclines induce and further exacerbate ferroptosis by producing excess iron as an initiating factor. GSH glutathione, GPX4 glutathione peroxidase 4, ROS reactive oxygen species, Tf transferrin, TfR transferrin receptor, PUFA polyunsaturated fatty acid, IRP iron response regulatory protein, IRE iron response element, Nrf2 nuclear factor erythroid 2‐related Factor 2, HMOX1 haem oxygenase 1, MitoFer mitochondria ferritin, ABCB8 ATP-binding cassette subfamily B member 8.

Ferroptosis is one of the essential forms in which anthracyclines exert their cytotoxic effects. Since ferroptosis is characterized by iron-dependent reactive oxygen species (ROS) deposition, anthracyclines can induce ferroptosis by increasing the pool of labile iron in cells [15], and lipid peroxides generated by ferroptosis are an indispensable source of excess ROS produced by the action of anthracyclines. The main mechanisms by which anthracyclines lead to iron disorders include mediating the upregulation of transferrin receptor (TfR) to allow more iron into the cell [16] and triggering the direct inactivation of ferritin and the indirect downregulation of ferritin due to the inactivation of iron regulatory proteins 1 and 2 (IRP1 and IRP2) [17]. Inactive IRPs bind to iron-response elements (IREs), modifying the expression of genes involved in iron metabolism. Anthracyclines also cause a decrease in ferritin by disrupting the mRNA of ferritin IRE [18]. Apart from these, in the mitochondria, anthracyclines elicit iron overload by blocking MitoFer and ABCB8. In the nucleus, activation of nuclear factor erythroid 2‐related Factor 2 (Nrf2) induces upregulation of haem oxygenase 1 (HMOX1), further resulting in the degradation of haem and the overproduction of free iron. Surplus iron complexed with anthracyclines releases ROS via the Fenton reaction, and anthracyclines further inhibit cytosolic and mitochondrial glutathione peroxidase 4 (GPX4) to drive lipid peroxidation, ultimately culminating in ferroptosis [19] (Fig. 1).

### Role and application of ferroptosis in anthracycline-related drug toxicity

#### Cardiotoxicity

**Fig. 2:**
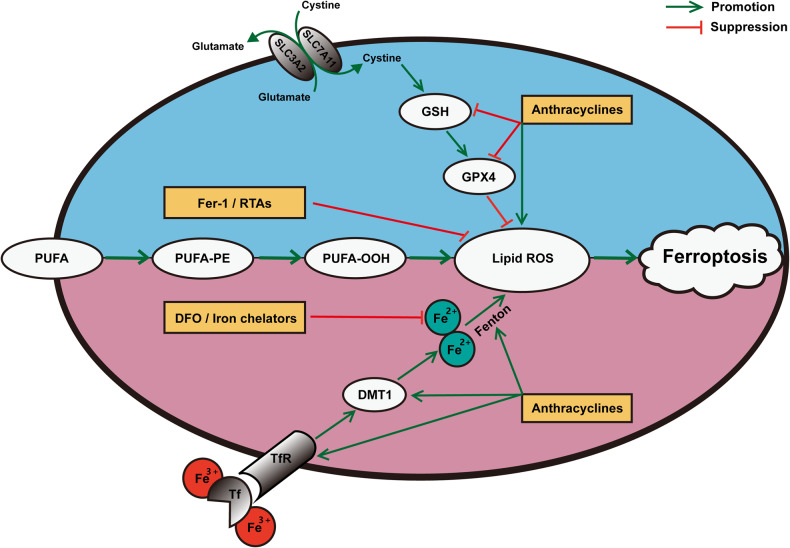
The pathogenesis and treatment of anthracycline-induced cardiotoxicity (AIC) based on ferroptosis. By means of ferroptosis, the pathogenesis of cardiotoxicity resulting from anthracyclines is that, on the one hand, anthracyclines directly mediate lipid peroxidation by affecting the levels of GSH, GPX4 and ROS, and on the other hand, the Fenton reaction is continuously triggered by their disruption of iron homeostasis. Correspondingly, the present drug development strategies to suppress AIC through the modulation of ferroptosis mainly involve iron chelators, such as DFO, to maintain iron homeostasis, and RTAs, such as Fer-1, to prevent lipid peroxidation. AIC anthracycline-induced cardiotoxicity, GSH glutathione, GPX4 glutathione peroxidase 4, ROS reactive oxygen species, DFO deferoxamine, RTAs radical-trapping antioxidants, Fer-1 ferrostatin-1, DMT1 divalent metal transporter 1, Tf transferrin, TfR transferrin receptor, PUFA polyunsaturated fatty acid, PUFA-PE polyunsaturated phosphatidylethanolamines.

**Fig. 3:**
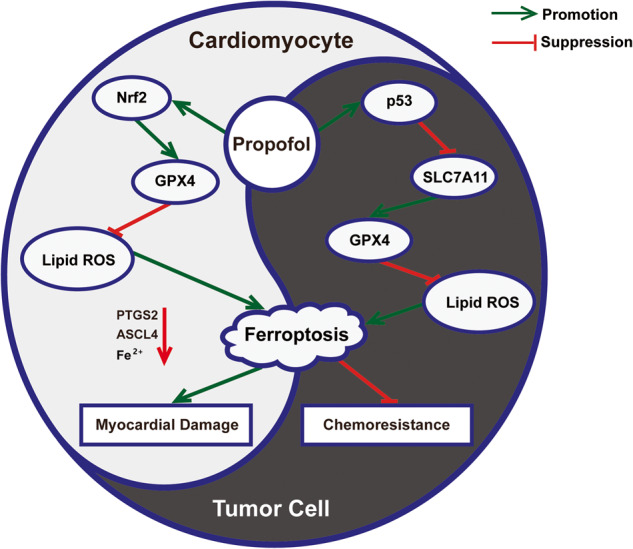
The dual effects of propofol on anthracyclines through ferroptosis. By activating the Nrf2/GPX4 and p53/SLC7A11 pathways, propofol can both inhibit lipid ROS in cardiomyocytes and elevate lipid ROS in tumour cells during anthracycline chemotherapy to achieve attenuated side effects and enhanced drug sensitivity. Nrf2 nuclear factor erythroid 2‐related Factor 2, GPX4 glutathione peroxidase 4, ROS reactive oxygen species, PTGS2 prostaglandin-endoperoxide synthase 2, ASCL4 achaete-scute complex-like 4.

Cardiotoxicity has long been recognized as the most serious side effect of anthracyclines following clinical administration [20]. Although oxidative stress was once thought to be the determining factor of the cardiotoxicity of anthracyclines [21], this classic view has been challenged as additional mechanisms have been subsequently demonstrated; among these mechanisms, ferroptosis is considered a particularly prominent one [3, 22]. It has been concluded that anthracyclines, such as DOX, induce ferroptosis in cardiomyocytes in the following two main ways (Fig. 2): on the one hand, they directly mediate lipid peroxidation by affecting the levels of glutathione (GSH), GPX4 and ROS, and on the other hand, the Fenton reaction is continuously triggered by their disruption of iron homeostasis [14]; based on these effects, multiple targets have been successively identified and strategies targeting these pathways have led to the development of significant interventions (Table 1) [23–42]. Among the mechanisms involved in these targets, the Nrf2 pathway accounts for a large proportion. Several plant-derived natural compounds, including astragaloside IV, fisetin, and resveratrol, have been proven to efficiently attenuate DOX-induced cardiotoxicity (DIC) by regulating ferroptosis; these effects are all closely related to the engagement of the Nrf2 pathway [36–38]. Moreover, Lu et al. [39] determined that propofol, which was protective in myocardial ischaemia‒reperfusion injury, also hindered DIC by mobilizing the Nrf2 pathway to inhibit ferroptosis in cardiomyocytes. Once activated, the Nrf2 pathway is thought to elevate downstream GPX4 levels to restrain lipid ROS production and decrease the expression of iron ions and ferroptosis-related genes, further antagonizing ferroptosis and protecting normal tissues (Fig. 3).

**Table 1:** Identified targets involved in ferroptosis related to anthracycline-induced cardiotoxicity (AIC).

Targets	Mechanism	Effect	Ref.
**Genes**			
Elabela	Upregulating KLF15/GPX4	Inhibiting ferroptosis and protecting cardiomyocytes	[23]
FUNDC2	Downregulating SLC25A11	Inducing ferroptosis and damaging cardiomyocytes	[24]
MITOL	Upregulating GPX4	Inhibiting ferroptosis and protecting cardiomyocytes	[25]
Acot1	Unclear	Inhibiting ferroptosis and protecting cardiomyocytes	[26]
PRMT4	Downregulating Nrf2/GPX4	Inducing ferroptosis and damaging cardiomyocytes	[27]
TRIM21	Unclear	Inducing ferroptosis and damaging cardiomyocytes	[28]
METTL14	Upregulating KCNQ1OT1/miR-7-5p	Inducing ferroptosis and damaging cardiomyocytes	[29]
FoxO4	Upregulating ENPP2	Inhibiting ferroptosis and protecting cardiomyocytes	[30]
Trx1	Upregulating mTORC1	Inhibiting ferroptosis and protecting cardiomyocytes	[31]
SPATA2	Upregulating CYLD	Inducing ferroptosis and damaging cardiomyocytes	[32]
HMOX1	Upregulating CTGF	Inducing ferroptosis and damaging cardiomyocytes	[33]
**Compounds**			
Epigallocatechin-3-gallate	Upregulating GPX4	Inhibiting ferroptosis and protecting cardiomyocytes	[34]
Salidroside	Upregulating GPX4	Inhibiting ferroptosis and protecting cardiomyocytes	[35]
Astragaloside IV	Upregulating Nrf2/GPX4	Inhibiting ferroptosis and protecting cardiomyocytes	[36]
Fisetin	Upregulating SIRT1/Nrf2/GPX4	Inhibiting ferroptosis and protecting cardiomyocytes	[37]
Resveratrol	Upregulating p62/Nrf2/GPX4	Inhibiting ferroptosis and protecting cardiomyocytes	[38]
Propofol	Upregulating Nrf2/GPX4	Inhibiting ferroptosis and protecting cardiomyocytes	[39]
LCZ696	Upregulating AKT/SIRT3/SOD2	Inhibiting ferroptosis and protecting cardiomyocytes	[40]
Melatonin	Upregulating YAP	Inhibiting ferroptosis and protecting cardiomyocytes	[41]
Histamine	Upregulating STAT3/SLC7A11	Inhibiting ferroptosis and protecting cardiomyocytes	[42]

Based on the corresponding toxicological mechanisms, the present drug development strategies to suppress AIC through the modulation of ferroptosis are mainly achieved by iron chelators that maintain iron homeostasis and radical-trapping antioxidants (RTAs) to prevent lipid peroxidation (Fig. 2) [14]. Dexrazoxane (DXZ), an iron chelator in the traditional sense, is the only cardioprotective agent approved by the U.S. Food and Drug Administration (FDA) for clinical purposes [43]. Although DXZ has been shown to reduce ferroptosis related to AIC, its cardioprotective effect is based on more than just the regulation of ferroptosis because it is not a specific ferroptosis inhibitor [14, 44]. Furthermore, the application of DXZ has faced diverse challenges, especially in children, as multiple pieces of evidence have shown that its effectiveness in reducing AIC in paediatric chemotherapy is not ideal [45, 46]. At the same time, the potential risk of aggravating the disease by eliciting secondary tumours due to DXZ is also worth noting [47]. Currently, the European Society of Cardiology (ESC) recommends DXZ only for patients with metastatic or advanced breast cancer at cumulative doses above 300 mg/m^2^ [48]. In addition, another iron chelator, deferoxamine (DFO), and a type of RTA, ferrostatin-1 (Fer-1), have been utilized in the treatment of AIC as definite suppressors of ferroptosis. DFO and Fer-1 protect cardiomyocytes from DOX by diminishing iron ions and decreasing lipid ROS, respectively, but strict dose control may be required during administration to guarantee efficacy and reduce side effects [49, 50]. Apart from these agents, although the remaining iron chelators and RTAs and novel drugs such as ethoxyquin [51] have the potential to relieve AIC on the basis of ferroptosis, their actual validity and safety are still doubtful [52].

#### Nephrotoxicity

The underlying nephrotoxicity of DOX has been known since the 1970s when Burke et al. initially reported a case of renal failure caused by DOX treatment [53]. Renal injury is not the primary side effect of DOX, and the relevant triggering mechanism is unclear and poorly studied; however, it has been suggested that it may involve iron dependence and be related to pathways such as oxidative stress and lipid peroxidation [54]. The nephrotoxicity of DOX has been increasingly recognized as possibly being associated with ferroptosis, with several studies showing a remarkable decrease in GSH levels and a significant increase in ROS levels in renal damage resulting from DOX [55, 56]. Qin et al. clarified the involvement of ferroptosis in DOX-induced nephrotoxicity through a series of in vivo and in vitro experiments; meanwhile, inhibition of ferroptosis could accordingly protect the kidney [57]. To date, astragaloside IV is the only substance that has been demonstrated to counteract the renal impairment of anthracyclines by modulating ferroptosis. Noticeably, for AIC, astragaloside IV also exerts considerable cardiac protection by the same ferroptosis mechanism that involves the activation of the Nrf2 signalling pathway [36, 57].

#### Hepatotoxicity

Liver injury arising from anthracyclines has been attributed to the ROS that are produced during drug metabolism in the liver [58]. Analogous to nephrotoxicity, anthracycline-mediated hepatotoxicity has not previously been a concern. Nevertheless, with the widespread adoption of third-generation anthracyclines such as pirarubicin and epirubicin, the potential side effects of liver damage are becoming more evident and of increasing concern, in addition to reducing cardiotoxicity compared to traditional DOX [59]. Shi et al. first identified that pirarubicin-induced hypohepatia was dependent on ferroptosis, and Fer-1, a ferroptosis inhibitor, significantly reversed the reduction in GPX4 and the elevation of ROS and total iron ions in impaired hepatocytes [60]. Apart from ferroptosis inhibitors, the classical agent schisandrin B has been characterized for its ability to attenuate pirarubicin-mediated hepatotoxicity by antagonizing ferroptosis, and its hepatoprotective effect is similarly regulated by the Nrf2 signalling pathway [60, 61].

### The role of ferroptosis in anthracycline-related tumour resistance

#### Triple-negative breast cancer

Currently, the potential targets against anthracycline-based chemoresistance that are involved in ferroptosis are mainly breast malignancies, haematologic malignancies, sarcomas, gynaecological tumours and drug-resistant cell lines (Table 2). Compared to other types of breast cancer, triple-negative breast cancer (TNBC) has limited therapeutic avenues, but it is extremely aggressive due to the lack of expression of human epidermal growth factor receptor-2 (HER2), progesterone receptor (PR) and oestrogen receptor (ER), often leading to a poor prognosis [62]. Anthracycline-based chemotherapy is a relatively monotonous but critically essential adjuvant therapy for TNBC patients, while the chemoresistance generated by TNBC often leaves these patients trapped in a treatment limbo [63]. A growing number of studies suggest that ferroptosis may be a breakthrough in solving this dilemma, and several have indicated that the efficacy of anthracyclines against TNBC and the occurrence of TNBC drug resistance are closely related to the level of RCD-regulated ROS [64–66]. Zhang et al. observed that by increasing GSH, TNBC cells that had undergone multiple iterations with DOX had fewer cytotoxic effects caused by the DOX-mediated rise in ROS [67], and the high level of GSH is now recognized as a crucial factor in the maintenance of DOX resistance in TNBC [68]. Recently, Sun et al. clarified that ferroptosis triggered by upregulation of iron content in the TNBC cell line MDA-MB-231 can make the cells more sensitive to DOX [69]. In addition, for TNBC cells, it was demonstrated that DNA dysfunction resulting from DOX treatment could supplement the ferroptosis mechanism to exert anticancer effects; this strategy has been applied in the development of novel targeted drugs [70].

**Table 2:** Potential targets involved in ferroptosis in relation to anthracycline-based chemoresistance.

Tumour type	Agent type	Sample type	Target	Mechanism/effect	Ref.
Triple-negative breast cancer	Doxorubicin	In vitro (MDA-MB-231 cells)	Isoliquiritin	Upregulation of isoliquiritin to induce ferroptosis and attenuate chemoresistance	[66]
Triple-negative breast cancer	Doxorubicin	In vivo (mice)	Phosphoglycerate dehydrogenase	Downregulation of phosphoglycerate dehydrogenase to reduce glutathione and attenuate chemoresistance	[67]
Triple-negative breast cancer	Doxorubicin	In vitro (MDA-MB-231 cells)	Glucose-6-phosphate dehydrogenase	Downregulation of glucose-6-phosphate dehydrogenase to reduce glutathione and attenuate chemoresistance	[68]
Acute myeloid leukaemia	Doxorubicin	In vitro (HL-60 cells)	p38α	Upregulation of p38α to induce ferroptosis and attenuate chemoresistance	[78]
Acute myeloid leukaemia	Daunorubicin	In vivo (mice)	Cystine	Downregulation of cystine to induce ferroptosis and attenuate chemoresistance	[79]
Diffuse large B-cell lymphoma	Doxorubicin	In vitro (a panel of 16 cell lines)	Ironomycin	Upregulation of ironomycin to induce ferroptosis and attenuate chemoresistance	[80]
Multiple myeloma	Doxorubicin	In vitro (H929 and RPMI-8226 cells)	Erastin	Upregulation of erastin to induce ferroptosis and attenuate chemoresistance	[81]
Uterine sarcoma	Doxorubicin	In vitro (MES-SA and FU-MMT-1 cells)	HSF1	Downregulation of HSF1 to induce ferroptosis and attenuate chemoresistance	[85]
Rhabdomyosarcoma	Doxorubicin	In vitro (U57810 and C2C12 cells)	ERK pathway	Upregulation of ERK pathway to induce ferroptosis and attenuate chemoresistance	[86]
Ovarian sarcoma	Doxorubicin	In vivo (mice)	Theanine	Upregulation of theanine to induce lipid peroxidation and attenuate chemoresistance	[89]
Osteosarcoma	Doxorubicin	Human tumour tissues	CBS, SOCS and EGFR	Downregulation of CBS and Upregulation of SOCS1 and EGFR to induce ferroptosis and attenuate chemoresistance	[103]
Cervical cancer	Doxorubicin	In vitro (HeLa and KB-V1 cells)	GSTM1 and GSTA1-3	Downregulation of GSTM1 and GSTA1-3 to reduce glutathione and attenuate chemoresistance	[97]
Ovarian cancer	Doxorubicin	In vitro (OV90 and SKOV3 cells)	Glutathione	Downregulation of glutathione to elevate reactive oxygen species and attenuate chemoresistance	[98, 99]
Drug-resistant cell line	Doxorubicin	In vitro (doxorubicin-resistant MES-SA/Dx5 sarcoma cells)	Glutathione	Downregulation of glutathione to elevate reactive oxygen species and attenuate chemoresistance	[106]
Drug-resistant cell line	Doxorubicin	In vitro (multidrug-resistant K562/ADM leukaemia cells)	AKT/mTOR pathway	Downregulation of AKT/mTOR pathway to induce ferroptosis and attenuate chemoresistance	[107]

#### Haematologic malignancies

Distinct from solid tumours, which can be treated with surgical resection and radiation therapy for masses, haematologic malignancies usually do not have a wide range of therapeutic options. Despite the successive emergence of advanced techniques such as relevant immunotherapy and targeted therapy, chemotherapy remains the cornerstone of haematologic tumour treatment [71, 72]. During leukaemia treatment, chemotherapy resistance to anthracyclines that are first-line regimens is considered to be associated with ferroptosis, and several studies have reported that the deleterious effect of DOX on the leukemic cell line CCRF-CEM can be significantly attenuated by ferroptosis inhibitors [73–76]. Correspondingly, in acute myeloid leukaemia (AML), a range of ferroptosis-inducing agents were shown to enhance the sensitivity of the promyelocytic leukaemia cell line HL-60 to DOX, and more crucially, they simultaneously augmented the anticancer activity of DOX [77, 78]. The combination of anthracyclines with ferroptosis-related antitumour agents always seems to lead to surprising outcomes in the treatment of haematologic disorders. The in vitro anti-leukemic activity of sulfasalazine was demonstrated to be achieved in part through the induction of ferroptosis by GSH reduction; among the eight types of anti-leukemic drugs, daunorubicin, which belongs to the anthracycline group, was identified as the best potentiator in combination with sulfasalazine [79]. In addition to leukaemia, Devin et al. [80] and Fu et al. [81] also found that there was a significant synergistic effect with ferroptosis inducers and DOX in diffuse large B-cell lymphoma (DLBCL) and multiple myeloma (MM), respectively. Moreover, DLBCL is the most common type of lymphoma, and its sensitivity to DOX has been confirmed to be classified depending on the level of ferroptosis-related gene expression [82].

#### Sarcomas

Sarcomas originating from mesenchymal tissue cover a vast array of subtypes, and the prominent heterogeneity among them can lead to differential prognosis [83]. Current therapies are ideal for only a small proportion of sarcomas, and chemotherapy regimens containing anthracyclines are undoubtedly the preferred adjuvant treatment route for chemotherapy-sensitive sarcomas but are generally ineffective for chemoresistant ones [84]. Han et al. established that a prognostic model constructed based on the expression of ferroptosis-related genes in uterine carcinosarcoma (UCS) patients was of substantial clinical value for predicting the chemosensitivity to anthracyclines. Subsequent knockdown of the ferroptosis suppressor gene heat shock Factor 1 (HSF1) in two uterine sarcoma cell lines confirmed that chemoresistance to DOX could be dramatically attenuated by enhanced ferroptosis [85]. Codenotti et al. revealed that treatment with diverse ferroptosis drivers could potentiate the lipid peroxidation response of rhabdomyosarcoma (RMS) in an extracellular signal-regulated kinase (ERK) pathway-dependent manner, and the efficacy of DOX was correspondingly improved in all treated RMS cell lines [86]. In the chemotherapy of multiple sarcomas, including osteosarcoma (OS), which is most common among adolescents, the diminished anticancer activity of anthracyclines has been shown to be correlated with reduced ROS levels and elevated GSH levels in tumour tissue [87–90]. In addition, ferroptosis can also promote the growth inhibitory effect of DOX on OS, along with hypoxic mechanisms [91].

#### Gynaecological oncology

In terms of gynaecological oncology, anthracyclines are concentrated in the chemotherapy of cervical cancer, endometrial cancer and ovarian cancer. Ferroptosis has been linked to the efficacy of relevant chemotherapies, and several studies have indicated that ferroptosis can be modulated to reverse the chemoresistance to varying degrees in all three malignancies [92]. Moreover, Gao et al. noted significant differences in chemotherapy outcomes between two groups of ovarian cancer patients in whom risk was classified based on ferroptosis-related long noncoding RNA (lncRNA) expression levels. In contrast to the high-risk group, the low-risk group presented with higher sensitivity and better efficacy to multiple chemotherapeutic agents [93]. Similarly, Li et al. [94] and Liu et al. [95] also identified the predictive value of ferroptosis-related gene expression levels for chemotherapy sensitivity in cervical cancer and endometrial cancer, respectively. In the management of cervical cancer, the reduction of ROS could attenuate the cytotoxicity of DOX [96], while the upregulation of GSH-related genes represented by glutathione S-transferases M1 and Alpha 1-3 (GSTM1 and GSTA1-3), enzyme activities and transporter proteins may be crucial factors leading to the development of DOX resistance in cervical cancer cells [97]. Similarly, in ovarian cancer, Manandhar et al. [98] and Shim et al. [99] also observed that elevated GSH levels contribute to a decreased sensitivity of tumour cells to anthracyclines, which is associated with the activation of the Nrf2 pathway. According to the latest research, dihydroartemisinin (DHA), as a common chemosensitizer, is equally suitable for DOX therapy in cervical cancer. The combination of DHA and DOX produced a highly synergistic lethal effect on cervical cancer cells by synergistic analysis; this effect was related to ferroptosis. In addition, DHA can also independently inhibit the proliferation of cervical cancer cells by ferroptosis, which is reflected in DHA-based induction of ferroptosis; this mechanism involves the accumulation of lipid ROS and the consumption of GPX4, which further exacerbates ferroptosis by promoting nuclear receptor coactivator 4 (NCOA4)-mediated ferritinophagy [100]. At present, although anthracyclines are widely applied in gynaecological malignancies, there are still few studies on drug resistance related to ferroptosis that need to be explored.

#### Drug-resistant cell lines

Despite the favourable efficacy of chemotherapy for certain tumours, once drug resistance occurs in originally chemotherapy-sensitive tumour cells, it usually heralds a poor prognosis [101]. As the most broadly deployed chemotherapeutic agent, the mechanism and resolution of anthracycline resistance has been a protracted clinical dilemma [102]. In previous studies, we observed that in OS cells with anthracycline resistance, the expression of suppressor of cytokine signalling 1 (SOCS1) and epidermal growth factor receptor (EGFR), which are considered drivers of ferroptosis, is notably downregulated, while the expression of cystathionine beta-synthase (CBS), which is considered a suppressor of ferroptosis, is significantly upregulated, and all three could be regarded as independent prognostic factors for OS patients [103]. Ferroptosis plays a vital role in clinical tumour drug resistance, and its inhibitor solute carrier family 7 member 11 (SLC7A11) is a promising target to overcome resistance in conventional cancer therapy. Blocking SLC7A11 can exert cytotoxic effects and reverse the low therapeutic sensitivity of cancer stem cells (CSCs) to DOX via a ferroptosis mechanism [104]. In addition, the expression of GPX4 was elevated in the DOX-resistant uterine sarcoma cell line MES-SA/Dx5 due to the overexpression of P-gp, while the decrease in GSH levels and the increase in ROS levels could re-enhance its chemosensitivity to DOX [105, 106]. Similarly, Zhang et al. identified that multidrug-resistant (MDR) leukaemia cells, including DOX-resistant cells, possess higher GSH expression than chemotherapy-sensitive cells, which may lead to a corresponding improvement in antioxidant capacity and buffering capacity against iron homeostasis dysregulation in initially sensitive cells to protect them from ferroptosis and prevent the development of drug resistance. This resistance can be reversed with ferroptosis-mediated inhibition of the AKT/mechanistic target of rapamycin (mTOR) pathway; thus, modulation of the iron homeostasis/ROS/AKT/mTOR pathway may be a potential strategy for the treatment of drug-resistant leukaemia [107]. Moreover, the induction of ferroptosis has now been demonstrated in multiple MDR cell lines involving breast cancer, colon cancer and glioblastoma, as a possible approach to sensitize these cell lines [73–76].

#### Cancer metastases

In addition to its widespread utilization in the aforementioned malignancies, intravenous anthracycline infusion, although no longer the preferred regimen in respiratory, gastrointestinal, and urologic cancers, is still appropriate and effective in patients with advanced metastases in these cancers, in which ferroptosis plays an essential role. Ferroptosis is recognized to be closely related to the mechanism of tumour metastasis [108]. It was revealed that elevated SLC7A11 expression due to H3K9me3 demethylation in the SLC7A11 promoter region prevented ferroptosis and further facilitated lung metastasis in OS cells [109]. SLC7A11 and downstream GPX4 are the core targets of the GSH-dependent antioxidant pathway, which has been identified as a central mechanism in the metastatic process of lung cancer [110], and harnessing these targets can lead to more effective chemotherapy strategies for patients with metastases. Liu et al. demonstrated that GPX4 inhibitors reduced chemoresistance in patients with brain metastases from non-small cell lung cancer [111]. Similarly, in the management of patients with gastric cancer metastasis, Jogo et al. found that downregulation of GPX4 expression could reverse the chemoresistance to increase the likelihood of promising results [112]. In addition, Drayton et al. observed in patients with advanced bladder cancer receiving systemic chemotherapy that cells with decreased SLC7A11 expression regained chemotherapy sensitivity in resistant patients [113]. In bladder cancer, lncRNA RP11-89 can repress ferroptosis in tumour cells to resist anthracycline cytotoxicity and is considered to be a pivotal element in the regulation of bladder cancer metastasis and treatment [114]. LncRNA RP11-89 upregulates prominin2 levels through the prominin2-multivesicular body (MVB)-exosome-ferritin pathway, and upregulated prominin2 further blocks intracellular iron accumulation through the MVB/exosome pathway to restrict ferroptosis [115]. In addition to primary metastases, ferroptosis plays an equally critical role in the management of secondary cancers. Liver metastasis is the most common cause of colorectal cancer, and the liver is a favourable site for ferroptosis. On the one hand, RCD mediated by lipid accumulation in hepatocytes is regarded as a potential contributor to liver tissue injury [116]. On the other hand, given that the liver is the main organ for iron deposition, the sensitivity of hepatocytes to ferroptosis is dramatically increased once the dysregulation of iron metabolism results in the generation of massive amounts of free iron [117]. Therefore, a reduction in the incidence of colorectal cancer can be achieved by specifically inhibiting ferroptosis to control liver tissue inflammation [118].

### Application of ferroptosis in anthracycline-related tumour resistance

#### Nanomedicines

**Fig. 4:**
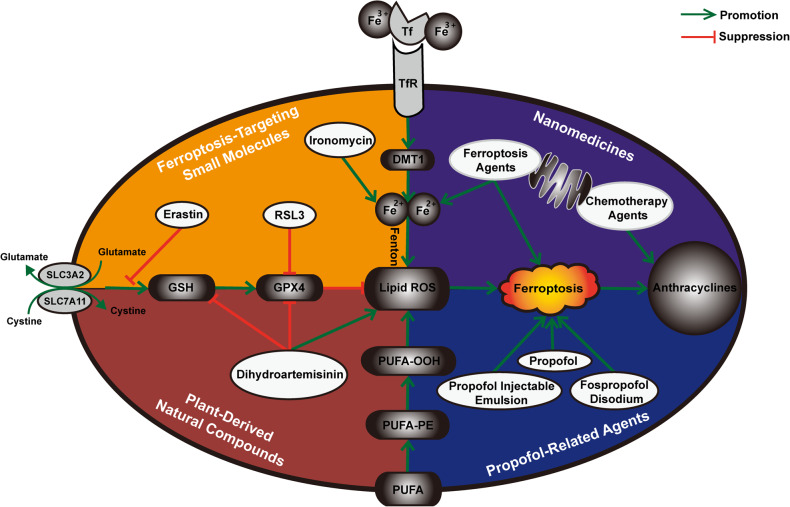
Strategies for modulating ferroptosis to overcome anthracycline-based chemoresistance. The main four specific areas include nanomedicines carrying both ferroptosis-inducing agents and anthracycline-based chemotherapeutic agents, traditional and novel ferroptosis-targeting small molecules, plant-derived natural compounds represented by dihydroartemisinin, and the recently discovered propofol-related drugs. GSH glutathione, GPX4 glutathione peroxidase 4, ROS reactive oxygen species, DMT1 divalent metal transporter 1, Tf transferrin, TfR transferrin receptor, PUFA polyunsaturated fatty acid, PUFA-PE polyunsaturated phosphatidylethanolamines.

To date, strategies to attenuate anthracycline-based chemoresistance by modulating ferroptosis primarily include nanomedicines, ferroptosis-targeting small molecules, plant-derived natural compounds and the recently discovered propofol-related drugs (Fig. 4). Nanomedicines carrying the active ingredients of chemotherapeutic agents are at the forefront of cancer therapy, and although their composition appears to radically differ, at the core, they all borrow the efficient target delivery of nanomaterials and the superior tumour tissue penetration of nanocarriers to enhance the efficacy of the corresponding chemotherapeutic agents. A series of nanomedicines using anthracyclines as carriers have been created and shown to significantly inhibit tumour proliferation while attenuating chemotherapeutic side effects [119]; the advent of ferroptosis has reinvigorated this series of drugs. Increasingly, novel nanomedicines are beginning to integrate diverse types of ferroptosis-inducing materials into the nanoplatform in addition to conventional anthracyclines during the synthesis process, and the ferroptosis effect mediated by these materials is defined to be synergistic with the apoptosis-based anticancer effect possessed by the anthracyclines; thereby, these strategies guarantee prominent clinical efficacy in the treatment of various cancers [120–122]. Furthermore, in recent reports, the ferroptosis mechanism could also collaborate with other RCD types, such as autophagy, to promote the cytotoxic effect of DOX [123]. Apart from the RCD pathway, nanomedicines can couple other pathways while mediating ferroptosis to antagonize tumour chemoresistance to anthracyclines. Fu et al. revealed that ferrate carried by nanomedicines triggered ferroptosis while simultaneously fostering an oxygen-rich environment and further downregulating the expression of hypoxia-inducible factor-1α (HIF-1α) and P-gp in OS cells, which reversed DOX resistance in OS [91]. The nanomedicine developed by Wang et al. that provoked ferroptosis was determined to additionally diminish the expression of ataxia telangiectasia mutated (ATM) to improve the sensitivity of prostate cancer cells to DOX [124]. Moreover, the fact that nanomedicines acting synergistically by virtue of DOX and ferroptosis also exist with the potential to increase the sensitivity of tumour cells to radiotherapy has been equally confirmed [125]. Despite the outstanding advantages of the relevant nanomedicines, how to ensure their high stability and effective biocompatibility remains a challenge that has to be faced today [126].

#### Ferroptosis-targeting small molecules

Erastin and RSL3, as traditional ferroptosis inducers whose mechanisms related to the regulation of ferroptosis are well defined, are not only widely utilized in clinical practice but also intimately associated with anthracycline chemoresistance. Erastin mediates ferroptosis by repressing system Xc- and is approved as an anticancer agent for the adjuvant treatment of several malignancies [127, 128]. Low doses of erastin have been demonstrated to enhance the anticancer activity of DOX and cytarabine, which are both front-line chemotherapeutic agents. Yu et al. identified that erastin substantially promoted the sensitivity of AML cells to anthracyclines in a non-RAS-dependent manner [78]. In addition, in vitro, erastin-triggered ferroptosis was reported to synergize with DOX to facilitate the overproduction of ROS for a combined tumour suppressive effect [81]. RSL3 mediates ferroptosis through inhibition of GPX4 and has exhibited underlying therapeutic value in a variety of cancers [129, 130]. A novel drug harbouring the RSL3 fragment developed by Nguyen et al. could boost the efficacy of anthracyclines by modulating the ferroptosis mechanism [131]. Pharmacologic combinations of RSL3 or erastin with DOX and actinomycin D were both validated to be effective in increasing cellular mortality in a variety of sarcoma cell lines [86]. Aside from traditional ferroptosis inducers, novel ferroptosis-targeting small molecules are emerging, most of which are synthetic derivatives of the original active compounds and have displayed their potential clinical value. A series of erastin derivatives not only improve the original potency of erastin but are also metabolically stable, and imidazole ketone erastin (IKE), the most distinguished derivative, has been characterized as a prominent anticancer agent in lung adenocarcinoma, fibrosarcoma and DLBCL [132–134]. In addition, ironomycin, an extremely promising small molecule substance synthesized on the basis of the natural product salinomycin, presents highly selective therapeutic activity against CSCs both in vivo and in vitro by specifically accumulating and sequestering lysosomal iron [135]. This effect has recently been clarified to correlate with the ferroptosis approach and to function more significantly therapeutically when combined with anthracyclines [80].

#### Plant-derived natural compounds

Plant-derived natural compounds tend to possess higher safety and fewer side effects in the treatment of tumours [136]. Artemisinin and its derivatives (ARTs) are of great interest as potential anticancer agents that trigger ferroptosis mainly by inhibiting the system Xc-/GPX4 pathway and enhancing lysosomal degradation of ferritin to elevate intracellular ROS levels [137]. A growing number of studies have indicated that ARTs are associated with chemoresistance, and ARTs have been validated to have significant therapeutic effects in various chemotherapy-resistant malignancies [138, 139]. In addition, Wang et al. revealed that low concentrations of artesunate counteracted the accumulation of chemoresistance in cancer cells [140]. DHA, as the active metabolite of ARTs, was also demonstrated to be closely linked to resistance to anthracyclines. Zhang et al. observed that DHA could enhance the chemosensitivity of DOX-resistant tumour cell lines by upregulating ROS expression and downregulating GPX4 and GSH expression, leading to the induction of ferroptosis and relying on DHA’s properties of facilitating oxidative damage and blocking antioxidant defence [107]. Moreover, ARTs have been reported to antagonize the ferroptosis effect by activating the Nrf2 pathway in the treatment of cisplatin-resistant head and neck cancer (HNC) cells; therefore, the combination of ARTs with Nrf2 inhibitors may improve efficacy [141, 142], but this has not been confirmed in relevant anthracycline studies. Apart from ARTs, multiple plant-derived natural compounds have been revealed to have potent cytotoxic effects on a range of anthracycline-resistant cell lines, which may be related to the regulation of ferroptosis mechanisms [73–76]. Nevertheless, the exact clinical value of these substances remains to be further ascertained.

#### Propofol

As one of the most frequently applied intravenous anaesthetics, propofol has shown additional efficacy in a multitude of other fields [143]. Especially concerning the pathogenesis and treatment of malignancies, it has been shown to be involved not only in epigenetic pathways but also in the regulation of various signalling pathways [144]. As mentioned in the previous section, propofol can attenuate the cardiomyocyte damage caused during anthracycline chemotherapy by modulating the ferroptosis mechanism [39]. Notably, it also possesses the potential to enhance the anticancer effect of anthracyclines, which interestingly may equally draw on the ferroptosis approach. Sun et al. revealed that propofol exerted antiproliferative action on TNBC cells and that three propofol-related agents, including propofol, fospropofol disodium and propofol injectable emulsion, all significantly amplified the efficacy of DOX in TNBC and that the RCD effects involved not only apoptosis but also ferroptosis mechanisms, including altered mitochondrial morphology, accumulated iron in tumour cells and elevated ROS levels, along with corresponding modification of the p53/SLC7A11 pathway [69] (Fig. 3). Consequently, it is reasonable to believe that propofol may be an enormously promising underlying chemotherapeutic adjuvant, but its specific efficacy and practical value remain to be verified.

#### Discussion and future perspectives

Ferroptosis is significantly associated with both tumour resistance and drug toxicity during anthracycline chemotherapy. From the perspective of tumour tissue alone, the activation of the ferroptosis effect can indeed suppress chemotherapy resistance to obtain higher anticancer efficacy, but from the perspective of normal tissue, only the silencing of the ferroptosis effect can inhibit chemotherapy toxicity to achieve lower side effects. Accordingly, it is necessary to integrate these two perspectives for further evaluation, whether in relevant studies or clinical applications. Lapatinib (LAP) is often applied clinically as an adjuvant in combination with anthracyclines because it can induce ferroptosis in tumour cells to attenuate chemotherapy resistance [145, 146]. Nevertheless, Sun et al. identified that LAP, despite enhancing the anticancer effect of DOX, also intensified the cardiotoxic effect of DOX equally by promoting ferroptosis in cardiomyocytes [147], which has been consistently overlooked before. Although DXZ is widely prescribed in combination with anthracyclines and is the only FDA-approved cardioprotective drug, it has never been considered whether the application of DXZ might reduce the efficacy of chemotherapy for tumours since its cardioprotective effect involves mediating the inhibition of ferroptosis. Meanwhile, it is also worth considering whether the previously reported deterioration of disease resulting from DXZ [47] is related to the suppression of ferroptosis. In addition, how can the pros and cons be weighed when clinically faced with the conflicting situation of having to exacerbate side effects to ensure the efficacy of chemotherapy or having to sacrifice part of the anticancer effect to protect normal target organs? All these issues remain to be further investigated and validated.

Chemotherapy is essentially a complex response that is systemically involved and encompasses various mechanisms, among which the pathways of chemotherapeutic toxicity caused by anthracyclines include inflammatory responses, calcium overload, ferroptosis, apoptosis and more [2], while the chemoresistance of tumours is determined by multiple factors, such as the tumour microenvironment, tumour heterogeneity and different RCD patterns [148, 149]. Notwithstanding the conflicting role that ferroptosis seems to play, ferroptosis mechanisms sometimes do not play a dominant or decisive role in the chemotherapeutic process. From another perspective, it is more noteworthy that the same substance may also induce dissimilar ferroptosis phenotypes in different target cells; for example, it was previously mentioned that propofol inhibits ferroptosis in cardiomyocytes by activating the Nrf2 pathway, whereas it enhances ferroptosis in tumour cells by modulating the p53/SLC7A11 pathway [39, 69]; this difference may be related to the triggering of different signalling pathways (Fig. 3). This may also provide insight for subsequent studies to elicit the desired ferroptosis phenotypes in different target cells using the corresponding signalling pathways for the same substance, with the aim of improving chemotherapeutic efficacy and alleviating side effects. According to our review, targeting the Nrf2 pathway in ferroptosis is a considerably valuable strategy to relieve anthracycline toxicities, including cardiotoxicity, nephrotoxicity and hepatotoxicity, all of which can be attenuated by the activation of the Nrf2 pathway; however, the synergistic effect of ferroptosis with the apoptosis-based anticancer effect of DOX has outstanding potential to address chemoresistance. This strategy has been demonstrated in diverse refractory tumours, including TNBC, sarcomas and MDR cancers. In addition to the utilization of corresponding pathways, currently, the development strategies of cutting-edge drugs are still mainly exploiting the high delivery efficiency of novel carriers, of which nanomedicines are the most typical, and exosome (EXO), a naturally low immunogenic substance, has also been recognized as a promising carrier for anthracyclines [150]. However, the majority of these novel agents are part of a passive approach to attenuate side effects, which often results from the specificity of the vector that allows a reduction in the amount of chemotherapy drugs for lower toxicity while ensuring efficacy.

In conclusion, ferroptosis is intimately related to anthracycline pharmacotherapy, and how to appropriately utilize the dual role played by ferroptosis and effectively avoid potential conflicts is core to the chemotherapy process. Targeting ferroptosis presents an eminent prospect for addressing both anthracycline-related tumour resistance and drug toxicity.
